# Supplementary material for: Reasons for non-attendance to cervical cancer screening and acceptability of HPV self-sampling among Bruneian women: A cross-sectional study
Source: PLoS One. 2022 Mar 14;17(3):e0262213. doi: 10.1371/journal.pone.0262213 (PMC8920207; doi:10.1371/journal.pone.0262213)
Supplement: S1 Table — (DOCX) [file pone.0262213.s001.docx]

S1 Table. Responses on their major and minor reasons for not attending cervical cancer screening among non-attendees at JPSHC, Brunei (Jan – Dec 2019)

| No. | Reasons for not attending screening | Major reason*  n (%) | Minor reason^  n (%) |
| --- | --- | --- | --- |
|  |  |  |  |
| 1 | I feel embarrassed being examined by a doctor or nurse | 26 (14.9) | 36 (20.7) |
| 2 | I am scared of pain because of previous bad experience(s) | 16 (9.2) | 13 (7.5) |
| 3 | I am afraid of getting a bad result | 28 (16.1) | 35 (20.1) |
| 4 | I can't find the time as I'm too busy at home | 18 (10.3) | 28 (16.1) |
| 5 | I can't find the time as I'm too busy at work | 16 (9.2) | 36 (20.7) |
| 6 | Nobody to send me to clinic | 2 (1.2) | 11 (6.3) |
| 7 | Nobody is looking after child(ren) at home | 2 (1.2) | 13 (7.5) |
| 8 | Difficult to get permission from employer | 8 (4.6) | 14 (8.1) |
| 9 | I have never heard of a Pap Test | 7 (4.0) | 8 (4.6) |
| 10 | I don't know what cervical cancer is | 1 (0.6) | 15 (8.6) |
| 11 | Not necessary as I am healthy | 4 (2.3) | 15 (8.6) |
| 12 | Not necessary as I am not child-bearing anymore | 4 (2.3) | 14 (8.0) |
| 13 | Not necessary as I don't have menses anymore | 4 (2.3) | 11 (6.3) |
| 14 | Not necessary as I don't have a partner anymore | 0 (0.0) | 1 (0.6) |
| 15 | Not necessary as I have already had my HPV vaccination | 2 (1.2) | 2 (1.2) |
| 16 | I have forgotten about it | 6 (3.4) | 2 (1.2) |
| 17 | I did not receive any invitation | 6 (3.4) | 0 (0.0) |
| 18 | Others | 15 (8.6) | 4 (2.3) |
|  | Missing | 9 (5.1) | 0 (0.0) |
|  | *One response for each participant |  |  |
|  | ^Multiple responses were allowed |  |  |
