# Supplementary material for: Reasons for non-attendance to cervical cancer screening and acceptability of HPV self-sampling among Bruneian women: A cross-sectional study
Source: PLoS One. 2022 Mar 14;17(3):e0262213. doi: 10.1371/journal.pone.0262213 (PMC8920207; doi:10.1371/journal.pone.0262213)
Supplement: S2 Table — Responses from the total study population (n = 174) were included. (DOCX) [file pone.0262213.s002.docx]

S2 Table. Attitudes towards cervical cancer screening among non-attendees at JPSHC, Brunei (Jan – Dec 2019). Responses from the total study population (n = 174) were included.

| No. | Attitude questions | Agree  n (%) | Neutral  n (%) | Disagree  n (%) |
| --- | --- | --- | --- | --- |
| 1 | I believe I am healthy and free of any diseases | 62 (35.6) | 61 (35.1) | 51 (29.3) |
| 2 | Having Pap test taken is beneficial for my health | 160 (92.0) | 10 (5.7) | 4 (2.3) |
| 3 | Like any women, I am susceptible to develop cervical cancer | 110 (63.2) | 52 (29.9) | 12 (6.9) |
| 4 | Cervical cancer can be severe and may lead to death | 144 (82.8) | 23 (13.2) | 7 (4.0) |
