# Supplementary material for: Reasons for non-attendance to cervical cancer screening and acceptability of HPV self-sampling among Bruneian women: A cross-sectional study
Source: PLoS One. 2022 Mar 14;17(3):e0262213. doi: 10.1371/journal.pone.0262213 (PMC8920207; doi:10.1371/journal.pone.0262213)
Supplement: S3 Table — (DOCX) [file pone.0262213.s003.docx]

S3 Table. Socio demographic characteristics and comparison between screening non-attendees who tested positive and negative for hr-HPV at JPSHC, Brunei (Jan – Dec 2019).

| Characteristics | | Total study population (n = 174) n (%) | Joined self-sampling  (n = 97) n (%) | hr-HPV positive  (n = 9) n (%) | hr-HPV negative  (n = 88) n (%) | p-value |
| --- | --- | --- | --- | --- | --- | --- |
| Median age in years (IQR) | | 45.0 (15.25) | 41.0 (17) | 35.0 (10) | 41.5 (16.5) | 0.115 |
| Age-group | 20 - 24 | 3 (1.7) | 3 (3.1) | 0 (0.0) | 3 (100) | 0.637 |
|  | 25 - 29 | 20 (11.5) | 15 (15.5) | 3 (20.0) | 12 (80.0) |  |
|  | 30 - 34 | 14 (8.1) | 10 (10.3) | 1 (10.0) | 9 (90.0) |  |
|  | 35 - 39 | 28 (16.1) | 18 (18.5) | 3 (16.7) | 15 (83.3) |  |
|  | 40 - 44 | 18 (10.3) | 11 (11.3) | 0 (0.0) | 11 (100) |  |
|  | 45 - 49 | 27 (15.5) | 12 (12.4) | 1 (8.3) | 11 (91.7) |  |
|  | 50 - 54 | 35 (20.1) | 15 (15.5) | 0 (0.0) | 15 (100) |  |
|  | 55 - 59 | 14 (8.1) | 10 (10.3) | 1 (10.0) | 9 (90.0) |  |
|  | > 60 | 13 (7.5) | 3 (3.1) | 0 (0.0) | 3 (100) |  |
|  | Missing | 2 (1.1) | 0 (0.0) | 0 (0.0) | 0 (0.0) |  |
| Race | Malay | 161 (92.5) | 90 (92.8) | 8 (8.9) | 82 (91.1) | 0.289 |
|  | Chinese | 6 (3.5) | 4 (4.1) | 0 (0.0) | 4 (100) |  |
|  | Other | 7 (4.0) | 3 (3.1) | 1 (33.3) | 2 (66.7) |  |
| Education level | Primary school | 16 (9.2) | 10 (10.3) | 2 (20.0) | 8 (80.0) | 0.456 |
|  | Secondary school | 96 (55.2) | 49 (50.5) | 4 (8.2) | 45 (91.8) |  |
|  | College / university | 57 (32.7) | 35 (36.1) | 3 (8.6) | 32 (91.4) |  |
|  | Missing | 5 (2.9) | 3 (3.1) | 0 (0.0) | 3 (100) |  |
| Marital status | Married | 157 (90.2) | 91 (93.8) | 9 (9.9) | 82 (90.1) | 1 |
|  | Divorced | 8 (4.6) | 2 (2.1) | 0 (0.0) | 2 (100) |  |
|  | Widowed | 9 (5.2) | 4 (4.1) | 0 (0.0) | 4 (100) |  |
| Occupation | Housewife | 64 (36.8) | 39 (40.2) | 5 (12.8) | 34 (87.2) | 0.862 |
|  | Government employee | 67 (38.5) | 41 (42.3) | 3 (7.3) | 38 (92.7) |  |
|  | Private employee | 31 (17.8) | 13 (13.4) | 1 (7.7) | 12 (92.3) |  |
|  | Retired | 9 (5.2) | 3 (3.1) | 0 (0.0) | 3 (100) |  |
|  | Unemployed | 1 (0.6) | 1 (1.0) | 0 (0.0) | 1 (100) |  |
|  | Other | 2 (1.1) | 0 (0.0) | 0 (0.0) | 0 (0.0) |  |
| Household income | < $500 | 27 (15.5) | 15 (15.5) | 1 (6.7) | 14 (93.3) | 0.612 |
|  | $500 < $999 | 27 (15.5) | 12 (12.4) | 0 (0.0) | 12 (100) |  |
|  | $1000-$1999 | 40 (23.0) | 22 (22.7) | 3 (13.6) | 19 (86.4) |  |
|  | $2000-$2999 | 19 (10.9) | 14 (14.3) | 0 (0.0) | 14 (100) |  |
|  | $3000-$5000 | 24 (13.8) | 15 (15.5) | 2 (13.3) | 13 (86.7) |  |
|  | >$5000 | 3 (1.7) | 2 (2.1) | 0 (0.0) | 2 (100) |  |
|  | Missing | 34 (19.5) | 17 (17.5) | 3 (17.6) | 14 (82.4) |  |
| No of births | 0 | 27 (15.5) | 17 (17.5) | 3 (17.6) | 14 (82.4) | 0.453 |
|  | 1 | 19 (10.9) | 14 (14.4) | 1 (7.1) | 13 (92.9) |  |
|  | 2 | 22 (12.1) | 14 (14.4) | 0 (0.0) | 14 (100) |  |
|  | 3 or more | 105 (60.3) | 51 (52.6) | 5 (9.8) | 46 (90.2) |  |
|  | Missing | 2 (1.2) | 1 (1.1) | 0 (0.0) | 1 (100) |  |
| Last Pap test done | Never | 41 (23.6) | 29 (29.9) | 4 (13.8) | 25 (86.2) | 0.313 |
|  | 4-10 years | 91 (52.3) | 48 (49.5) | 4 (8.3) | 44 (91.7) |  |
|  | > 10 years | 36 (20.7) | 15 (15.5) | 0 (0.0) | 15 (100) |  |
|  | Missing | 6 (3.4) | 5 (5.1) | 1 (20.0) | 4 (80.0) |  |
| HPV vaccination status | Unvaccinated | 95 (54.6) | 50 (51.6) | 6 (12.0) | 44 (88.0) | 0.899 |
|  | Fully vaccinated | 27 (15.5) | 16 (16.5) | 1 (6.3) | 15 (93.7) |  |
|  | Partly vaccinated | 41 (23.6) | 26 (26.8) | 2 (7.7) | 24 (92.3) |  |
|  | Missing | 11 (6.3) | 5 (5.1) | 0 (0.0) | 5 (100) |  |
