# Supplementary material for: Reasons for non-attendance to cervical cancer screening and acceptability of HPV self-sampling among Bruneian women: A cross-sectional study
Source: PLoS One. 2022 Mar 14;17(3):e0262213. doi: 10.1371/journal.pone.0262213 (PMC8920207; doi:10.1371/journal.pone.0262213)
Supplement: S1 Fig — The x-axis indicates the percentage, and the number next to each bar indicates the number of responses. Multiple responses were allowed and responses from the total study population (n = 174) were included. (DOCX) [file pone.0262213.s004.docx]

S1 Fig. Preferred sources of information about cervical cancer among non-attendees at JPSHC, Brunei (Jan–Dec 2019). The x-axis indicates the percentage, and the number next to each bar indicates the number of responses. Multiple responses were allowed and responses from the total study population (n = 174) were included.
