# Supplementary material for: Reasons for non-attendance to cervical cancer screening and acceptability of HPV self-sampling among Bruneian women: A cross-sectional study
Source: PLoS One. 2022 Mar 14;17(3):e0262213. doi: 10.1371/journal.pone.0262213 (PMC8920207; doi:10.1371/journal.pone.0262213)
Supplement: S1 Questionnaire — (DOCX) [file pone.0262213.s005.docx]

| **Borang Soal Selidik 1:**  ***Kajian Untuk Mengetahui Sebab-Sebab Kenapa Wanita Tidak Mengikuti Program Penyaringan Kanser Servik Kebangsaan***  **Questionnaire 1:**  **Reasons why women do not participate *in the National Cervical Cancer Screening Programme***  **Tujuan kajian ini:**  Kajian ini dijalankan bagi memahami sebab-sebab wanita di Negara Brunei Darussalam tidak mengikuti Program Penyaringan Kanser Serviks Kebangsaan. Penemuan dari kajian ini diharap akan dapat membantu Kementerian Kesihatan untuk merancang strategi-strategi yang lebih berkesan bagi meningkatkan liputan penyaringan kanser servik di Negara Brunei Darussalam.  **Untuk responden:**  Sila jawab **SEMUA** soalan-soalan.  Jawapan Dayang hanya akan digunakan untuk tujuan kajian ini dan semua maklumat peribadi akan dirahsiakan.  ***Aim of this study:***  *This survey is designed to understand the reasons why women do not participate in the National Cervical Cancer Screening Programme. Findings from this study will help the Ministry of Health to plan effective strategies for increasing cervical screening coverage in Brunei Darussalam.*  ***For respondents:***  *Please answer ALL questions. Your responses will be used only for the purpose of this study and personal information will be kept confidential.* |
| --- |

PIN no:


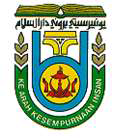

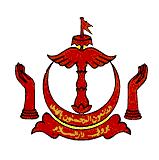


**INSTITUT SAINS KESIHATAN PAPRSB PAPRSB INSTITUTE OF HEALTH SCIENCES**

**UNIVERSITI BRUNEI DARUSSALAM**

**KEMENTERIAN KESIHATAN**

**MINISTRY OF HEALTH**

**BRUNEI DARUSSALAM**

***Penyertaan Dayang adalah sangat-sangat dihargai***

***Your participation is greatly appreciated***

| **MAKLUMAT DEMOGRAFI RESPONDEN**  **RESPONDENT’S DEMOGRAPHIC DETAILS** | | | | | | | | |
| --- | --- | --- | --- | --- | --- | --- | --- | --- |
| **Umur**  *Age* |  | | | | | | | |
| **Bangsa**  *Race* | ☐ Melayu  *Malay* | | | ☐ Cina  *Chinese* | | | ☐ India  *Indian* | |
|  | ☐ Lain-lain, sila nyatakan:  *Others, specify:* | | | | | | | |
| **Warna IC**  *IC colour* | ☐ Kuning  *Yellow* | | | | ☐ Merah  *Red* | | | |
| **Kelulusan Pengajian Tertinggi** *Highest education attained* | ☐ Sekolah Rendah  *Primary school* | | ☐ Sekolah *Menengah*  *Secondary school* | | | ☐ Kolej/Universiti  *College /University* | | |
| **Taraf Kelamin**  *Marital Status* | ☐ Kahwin  *Married* | | | ☐ Janda  *Divorced* | | | ☐ Balu  *Widowed* | |
| **Pekerjaan**  *Occupation* | ☐ Pekerja Kerajaan *Government employee* | | | ☐Pekerja Swasta  *Private Employee* | | | ☐ Penuntut  *Student* | |
|  | ☐ Surirumah  *Housewife* | | | ☐ Bersara  *Retired* | | | ☐Menganggur  *Unemployed* | |
| **Purata Pendapatan**  **Keluarga Sebulan**  *Average monthly income*  *for the household* | ☐ < $500 | | | ☐ $500 - $1000 | | | ☐ $1000 - B$2000 | |
|  | ☐ $2000 - $3000 | | | ☐$3000 - B$5000 | | | ☐ > B$5000 | |
| **Pernahkah Dayang memperolehi TIGA dos suntikan HPV?**  *Have you received all THREE doses of HPV vaccination?* | ☐ Ya  *Yes* | | | ☐ Tidak  *No* | | | ☐ Belum pernah  *Never* | |
| **Adakah Dayang mengandung**  **pada masa ini?** *Are you currently pregnant?* | ☐ Ya  *Yes* | | | ☐ Tidak  *No* | | | ☐ Kurang pasti  *Not sure* | |
| **Jumlah Kelahiran**  *Number of births* | ☐ 0 | ☐ 1 | | | ☐ 2 | | | ☐ 3 atau lebih  *3 or more* |
| **Ujian Pap Kali Terakhir**  *Last Pap test done* | ☐ Tidak Pernah  *Never* | ☐Sebelum 2007  *Before 2007* | | | ☐ 2008 - 2010 | | | ☐ 2011 - 2013 |

| **Please mark "1"** next to your **MAIN** reason | **Sebab-sebab tidak mendapatkan ujian Pap dalam tempoh 4 tahun kebelakangan**  **(**Dayang boleh memilih **lebih dari satu jawapan)**  ***Your reasons for not getting a Pap test in the previous 4 years***  *(You may choose* ***more than one answer****)* |
| --- | --- |
|  | ☐Merasa malu diperiksa oleh doktor atau jururawat  *I feel embarassed being examined by a doctor or nurse* |
|  | ☐Takut sakit disebabkan pengalaman buruk dahulu  *I am scared of pain because of previous bad experience(s)* |
|  | ☐Takut mendapat keputusan yang tidak normal  *I am scared of getting a bad result* |
|  | ☐Tidak ada masa kerana terlalu sibuk di rumah  *I can't find the time as I'm too busy at home* |
|  | ☐Tidak ada masa kerana terlalu sibuk di pejabat  *I can't find the time as I'm too busy at work* |
|  | ☐Tidak ada orang yang dapat menghantar saya ke klinik  *Nobody to send me to clinic* |
|  | ☐Tidak ada orang menjaga anak-anak saya di rumah  *Nobody is looking after child(ren) at home* |
|  | ☐Sukar untuk mendapat kebenaran keluar pejabat  *Difficult to get permission from employer* |
|  | ☐Tidak pernah mendengar mengenai Ujian Pap  *I have never heard of a Pap Test* |
|  | ☐Tidak tahu apa itu kanser servik/ kanser pangkal rahim  *I don't know what cervical cancer is* |
|  | ☐Tidak perlu kerana saya merasa sihat  *Not necessary as I am healthy* |
|  | ☐Tidak perlu kerana saya tidak lagi melahirkan anak  *Not necessary as I am not child-bearing anymore* |
|  | ☐Tidak perlu kerana saya sudah putus haid ( menopos)  *Not necessary as I don't have menses anymore* |
|  | ☐Tidak perlu kerana saya tidak lagi mempunyai pasangan  *Not necessary as I don't have a partner anymore* |
|  | ☐Tidak perlu kerana saya sudah menerima suntikan HPV  *Not necessary as I have already had my HPV vaccination* |
|  | ☐Tidak perlu kerana saya tidak lagi mempunyai rahim selepas pembedahan  *Not necessary as I've had surgery ( hysterectomy) to remove my uterus* |
|  | ☐Lain-lain, sila nyatakan:  *Others, please specify:* |

Please mark "1" next to your **MAIN** reason

| **Sikap/Attitude** | | | | | | | | |
| --- | --- | --- | --- | --- | --- | --- | --- | --- |
| 1. **Saya percaya bahawa saya sihat dan tidak mempunyai sebarang penyakit**   ***I believe that I am healthy and free of any disease*** | | | | | | | | |
| ☐ Sangat Setuju  *Strongly agree* | ☐Setuju  *Agree* | | | ☐ Tiada pendapat  *Neutral* | ☐ Tidak setuju  *Disagree* | | | ☐ Sangat tidak setuju  *Strongly disagree* |
| 1. **Menjalani ujian Pap adalah bermanfaat buat kesihatan saya**   ***Having a Pap test taken is beneficial for my health*** | | | | | | | | |
| ☐ Sangat Setuju  *Strongly agree* | ☐Setuju  *Agree* | | | ☐ Tiada pendapat  *Neutral* | ☐ Tidak setuju  *Disagree* | | | ☐ Sangat tidak setuju  *Strongly disagree* |
| 1. **Seperti wanita lain, saya juga boleh mendapat kanser servik**   ***Like any women, I am susceptible to develop cervical cancer*** | | | | | | | | |
| ☐ Sangat Setuju  *Strongly agree* | ☐Setuju  *Agree* | | | ☐ Tiada pendapat  *Neutral* | ☐ Tidak setuju  *Disagree* | | | ☐ Sangat tidak setuju  *Strongly disagree* |
| 1. **Kanser servik boleh menjadi teruk dan mengakibatkan kematian**   ***Cervical cancer can be severe and may lead to death*** | | | | | | | | |
| ☐ Sangat Setuju  *Strongly agree* | ☐Setuju  *Agree* | | | ☐ Tiada pendapat  *Neutral* | ☐ Tidak setuju  *Disagree* | | | ☐ Sangat tidak setuju  *Strongly disagree* |
| **Maklumat / Information** | | | | | | | | |
| 1. **Adakah Dayang ingin memperolehi maklumat lanjut mengenai Ujian Pap?**   ***Would you like to get more information about Pap test?*** | | | | | | | | |
| ☐ Ya  *Yes* | | | ☐ Tidak  *No* | | | ☐ Tidak tahu  *Don't know* | | |
| 1. **Dari mana atau dari siapa Dayang ingin mendapatkan maklumat mengenai ujian Pap?**   (Dayang boleh memilih lebih dari satu jawapan)  ***Where or from whom would you like to get more information about the Pap Test?***  *(You may choose one or more answers)* | | | | | | | | |
| ☐ Radio | | ☐ Pekerja Kesihatan  *Healthcare worker* | | | | | ☐ SMS / WhatsApp | |
| ☐ Televisyen  *Television (RTB)* | | ☐Laman Sesawang Kementerian Kesihatan  *Ministry of Health’s website* | | | | | ☐ Poster/Sepanduk  *Posters/ Banners* | |
| ☐ Suratkhabar  *Newspapers* | | ☐ Media Sosial  *Social media (e.g. Facebook)* | | | | | ☐ Tidak tahu  *Don't know* | |
| ☐Lain-lain, sila nyatakan:  *Others, please specify:* | | | | | | | | |

***Tamat. Terima Kasih atas bantuan Dayang!***

***End of Questionnaire. Thank you very much for your cooperation!***
