# Supplementary material for: Reasons for non-attendance to cervical cancer screening and acceptability of HPV self-sampling among Bruneian women: A cross-sectional study
Source: PLoS One. 2022 Mar 14;17(3):e0262213. doi: 10.1371/journal.pone.0262213 (PMC8920207; doi:10.1371/journal.pone.0262213)
Supplement: S2 Questionnaire — (DOCX) [file pone.0262213.s006.docx]

| **Borang Soal Selidik 2:**  **"Penerimaan Pengambilan Sampel Sendiri bagi Ujian HPV di Kalangan Wanita-Wanita yang Tidak Mengikuti Program Penyaringan Kanser Servik di Negara Brunei Darussalam”**  **Questionnaire 2:**  "**Acceptability of Self Sampling for HPV Testing Among Non-attendees of Cervical Screening Programme in Brunei Darussalam**"  (to be completed AFTER self-sampling)  **Tujuan kajian ini:**  Kajian ini dijalankan bagi memahami sebab-sebab wanita di Negara Brunei Darussalam tidak mengikuti Program Penyaringan Kanser Serviks Kebangsaan. Penemuan dari kajian ini diharap akan dapat membantu Kementerian Kesihatan untuk merancang strategi-strategi yang lebih berkesan bagi meningkatkan liputan penyaringan kanser servik di Negara Brunei Darussalam.  **Untuk responden:**  Sila jawab **SEMUA** soalan-soalan.  Jawapan Dayang hanya akan digunakan untuk tujuan kajian ini dan semua maklumat peribadi akan dirahsiakan.  ***Aim of this study:***  *This survey is designed to understand what local women think about the HPV self-sampling for HPV testing. Findings from this study will help the Ministry of Health to determine if such self-sampling would be an effective approach to increase cervical screening coverage in Brunei Darussalam.*  ***For respondents:***  *Please answer ALL questions. Your responses will be used only for the purpose of this study and personal information will be kept confidential.* |
| --- |


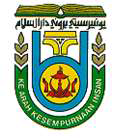

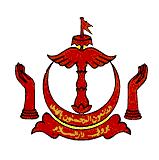


PIN no:

***Penyertaan Dayang adalah sangat-sangat dihargai***

***Your participation is greatly appreciated***

**INSTITUT SAINS KESIHATAN PAPRSB PAPRSB INSTITUTE OF HEALTH SCIENCES**

**UNIVERSITI BRUNEI DARUSSALAM**

**KEMENTERIAN KESIHATAN**

**MINISTRY OF HEALTH**

**BRUNEI DARUSSALAM**

Borang Soal Selidik 2 bagi:

**"Penerimaan Pengambilan Sampel Sendiri bagi Ujian HPV di Kalangan Wanita-Wanita yang Tidak Mengikuti Program Penyaringan Kanser Servik di Negara Brunei Darussalam”**

*Questionnaire 2 for:*

*"****Acceptability of Self Sampling for HPV Testing Among Non-attendees of Cervical Screening Programme in Brunei Darussalam****"*

1. Sila baca kenyataan yang diberikan.

*Please read the statements given below.*

1. Gunakan skala yang disediakan bagi menyatakan pendapat Dayang

*Please use the scale provided to state your opinion*

1. Sila tandakan (√) dalam petak berkenaan.

*Please tick the appropriate boxes.*

**PANDUAN SKALA / *SCALE GUIDE***

1 = Sangat Tidak Setuju / *Strongly disagree*

2 = Tidak Setuju / *Disagree*

3 = Tidak Pasti / *Unsure*

4 = Setuju/ *Agree*

5 = Sangat Setuju/ *Strongly agree*

| **PERKARA**  **ITEM** | **Sangat tidak setuju**  *Strongly disagree* | **Tidak setuju**  *Disagree* | **Tidak Pasti**  *Unsure* | **Setuju**  *Agree* | **Sangat Setuju**  *Strongly agree* |
| --- | --- | --- | --- | --- | --- |
|  | **1** | **2** | **3** | **4** | **5** |
| Arahan cara pengambilan contoh mudah difahami  *I thought the instructions were clear* |  |  |  |  |  |
| Pengambilan contoh adalah mudah  *It was easy to do the swab* |  |  |  |  |  |
| Pengambilan contoh menyakitkan saya  *Taking the sample with the swab was painful* |  |  |  |  |  |
| Pengambilan contoh adalah tidak selesa  *Taking the sample was uncomfortable to do* |  |  |  |  |  |
| Saya merasa malu melakukan pengambilan sendiri  *I felt embarassed doing the self-sampling* |  |  |  |  |  |
| **PERKARA**  **ITEM** | **Sangat tidak setuju**  ***Strongly disagree*** | **Tidak setuju**  ***Disagree*** | **Tidak Pasti**  ***Unsure*** | **Setuju**  ***Agree*** | **Sangat Setuju**  ***Strongly agree*** |
|  | **1** | **2** | **3** | **4** | **5** |
| Pengambilan contoh sendiri amat memudahkan saya tanpa perlu menjalani Ujian Pap  *It was convenient to do without having to undergo a Pap Test* |  |  |  |  |  |
| Saya yakin saya melakukannya dengan betul  *I am confident I did it correctly* |  |  |  |  |  |
| Saya mahu menggunakan cara pengambilan ini di masa akan datang  *I want to use this method next time* |  |  |  |  |  |
| Saya lebih suka melakukan pengambilan contoh ini di rumah  *I prefer to do this at home* |  |  |  |  |  |
| Saya akan mensyorkan wanita lain untuk mencuba cara ini  *I would recommend this method to other women* |  |  |  |  |  |
| Saya percaya keputusan dari pengambilan sendiri ini adalah tepat  *I trust that the result of this self-sampling will be accurate* |  |  |  |  |  |
| Saya mahu menjalani Ujian Pap yang sebenarnya di klinik di masa akan datang  *I would like to attend for a proper Pap Test in clinic next time* |  |  |  |  |  |

***Tamat. Terima Kasih atas bantuan Dayang!***

***End of Questionnaire. Thank you very much for your cooperation!***
