# Supplementary material for: Reasons for non-attendance to cervical cancer screening and acceptability of HPV self-sampling among Bruneian women: A cross-sectional study
Source: PLoS One. 2022 Mar 14;17(3):e0262213. doi: 10.1371/journal.pone.0262213 (PMC8920207; doi:10.1371/journal.pone.0262213)
Supplement: Attachment — Submitted filename: Response to Reviewers.docx [file pone.0262213.s008.docx]

**Point-to-point responses:**

| From the Journal | Author’s Response |
| --- | --- |
| 1. Please ensure that your manuscript meets PLOS ONE's style requirements, including those for file naming. | The manuscript has been revised as requested, including the use of American English. |
| 2. Please provide additional details regarding participant consent. In the ethics statement in the Methods and online submission information, please ensure that you have specified what type you obtained (for instance, written or verbal, and if verbal, how it was documented and witnessed). If your study included minors, state whether you obtained consent from parents or guardians. If the need for consent was waived by the ethics committee, please include this information. | Participants gave written consent for both parts of the study. We have added this point in the Methods section (3rd paragraph under Study design and data collection sub-section):  “Participants who gave written consent to the first part of the study were asked to complete the first questionnaire (Q1) onsite. They were then given an envelope containing information on the second part and an instruction leaflet on the HPV self-sampling procedure. Upon second contact about two weeks later, those who gave verbal consent were given an appointment to JPSHC. On the day, a trained nurse first explained the procedure using an instructional video and answered any questions. Participants who gave another written consent were then given a self-sampling kit and asked to perform the procedure in the clinic. After completion, they were asked to complete the second questionnaire (Q2).” |
| i3. Please include additional information regarding the survey or questionnaire used in the study and ensure that you have provided sufficient details that others could replicate the analyses. For instance, if you developed a questionnaire as part of this study and it is not under a copyright more restrictive than CC-BY, please include a copy, in both the original language and English, as Supporting Information. | Both questionnaires used in this study are now attached as Supplementary Information (S4 & S5). |
| 4. In your Data Availability statement, you have not specified where the minimal data set underlying the results described in your manuscript can be found. PLOS defines a study's minimal data set as the underlying data used to reach the conclusions drawn in the manuscript and any additional data required to replicate the reported study findings in their entirety. All PLOS journals require that the minimal data set be made fully available. | We have attached the minimal dataset as Supplementary Information (S6). |

| Comments from the reviewers | Author’s Response |
| --- | --- |
| Reviewer #1: The authors have undertaken a questionnaire based small survey in Brunei women about attitude towards cervical screening. Background is that Brunei has a high incidence of cervical cancer and a low screening coverage of 44%.  The results are worth informing the world about. But the manuscript is fair too long for what it contains. It should be shortened considerably:  1. delete most of page 6 2. Delete figure 2 3. Merge Tables 3 and 4 4. Delete figure 3 | Thank you for your comments. We have edited the whole manuscript to correct any grammatical mistakes and also make our points shorter and more concise.  As suggested, we have also:  1. Shorten the data collection method in page 6.  2. Merged Tables 3 and 4, whereby the total count column was added into the original Table 4. The original Table 3 is now moved to the Supplementary Information (as Table S2), for readers who want to refer to the breakdown responses (agree, neutral & disagree).  3. Figure 3 was also moved to the Supplementary Information (as Figure S1), for readers who want to know the full breakdown of responses.  For Figure 2, we decided to keep Figure 2 because we think it is the best way to summarize both counts & percentages for both major and minor reasons (and showing the complete picture of our study results). Table S1 shows the same results in the Table version, but it is less intuitive as Figure 2. Also, Table 2 only show the results for the top 10 major reasons (meaning that it is not showing the complete picture, like Figure 2). |
| 5. Shorten considerable the three points in the Discussion – really to about two lines each I miss Discussion about:  1. why is the mean age so high 45 years? 2. Screening coverage is really not so low in participants: 52.3% had been screening within last 4-10 years 3. How does the reported screening coverage in the group correspond to the national coverage of 44%? 4. By for the majority of women answer agree to “having a pap test taken is beneficial for my health” - when why do the authors recommend more education and awareness as their top priority? - women actually seem to be fairly good informed. 5. More comments should be given on potential practical and cultural barriers. | 5.1. The median age of 45 years could be due to the specific population that we have targeted, that is, the screening non-attendees. As mentioned in the Methods section: “The inclusion criteria were married or ever married Bruneian women between 20 and 65 years old, who either never or did not have a Pap test within the last 4 years, and who can comprehend Brunei-Malay or English language”.  While it is possible this could be due the convenience sampling that were conducted in this study, we take note that previous studies from Finland (Virtanen A et al. Ref no.18 in manuscript) and El Salvador (Maza M, et al. Ref no.5 in manuscript), both focusing on screening non-attendees only, also have similar median ages. Although both studies include women between 30-60 years old, our study is still comparable with theirs as the median age of marriage in Brunei is about 26 years from 2014 to 2019 (based on country census data: <http://www.deps.gov.bn/SitePages/Vital%20Statistics.aspx> )  In the revised manuscript, we decided not to explicitly include this point. Instead, we have added a new paragraph on the potential cultural barriers (please see item 5.5 below) which elude to this point.    5.2. Thank you for your point here. In this study, we want to investigate reasons for both non-attendance to screening at all, as well as for not continuing with screening every 3 years, as recommended. This is why women who had undergone screening within the last 4-10 years were included in this study. To clarify this point and avoid confusion, we have replaced the word “coverage” to “uptake” in our study significance statement (Introduction section, last sentence): *“Study findings could be used to strategize ways to improve screening* ***uptake*** *and provide preliminary evidence towards implementing HPV testing as the primary screening test for cervical cancer in Brunei.”*  5.3. This point relates to the sampling strategy for this study. Briefly, our study findings could not be generalizable to the whole country population. Please see our response for Reviewer #2 below, who commented on the result generalizability.  5.4. We would like to clarify that we are pushing for promoting knowledge on cervical cancer, the benefits of screening and clarifying any misconceptions of Pap test results. This implies not only on promoting general awareness on the Pap test, but also on why is this important to be conducted periodically and to clarify any misconceptions of the Pap test results. To prevent any misunderstanding, the conclusion paragraph is revised as follows:  *“In conclusion, our findings indicate the need to further promote knowledge on cervical cancer, the benefits of screening and clarifying any misconceptions of Pap test results. Reasons of cervical cancer screening non-attendance were mainly related to emotional and logistical factors. As we found high acceptance towards HPV self-sampling, this could be adopted as an alternative for women who refrain from Pap test. Our high detection of non-16/18 HPV genotypes suggest high prevalence of other hr-HPV genotypes in Brunei. Future larger studies involving more Bruneian women should be done to verify our results. Follow-up studies should also be conducted to consider HPV testing as the suitable method for cervical cancer screening.”*  5.5. Thank you for your point here. We have added a new paragraph on the potential cultural barriers:  *“One notable point for the local context relates to the presence of cultural barriers. Being a predominantly Islamic society, religious and cultural modesty could be a contributing factor for embarrassment among Muslim women [39]. Also, having premarital sex is a cultural taboo in Brunei and is generally not openly discussed [40]. This could prevent any unmarried but sexually active women from participating in the screening program. It should be emphasized that only married or ever married females were included in this study; included because it is part of the eligibility criteria for the national cervical cancer screening program in Brunei.”* |
| Overall: relevant topic, but not acceptable for publication in its present form. Should be reduced to a max. Of haft the number of words to be of interest for a broader audience. | Thank you for your comment. We have edited the manuscript and have reduced the word count to 3049 words (manuscript, excluding references). We hope this revision would be clear and concise enough for the journal readers. |
| Reviewer #2: This is an important topic. Please consider how you can make these results of 174 women generalizable to the entire Brunei population. How do you exclude sampling bias? Why did you do this in two parts? | Thank you for your comments.  (a) and (b). It will be difficult to generalize the study results, as there was only one study site, sample size was low & sampling bias was done conveniently. We have emphasized this in the limitations:  “*A major limitation for this study is that non-attendees from only one health center were recruited, thus our findings are not representative of the adult female population in Brunei. Secondly, our findings should also be interpreted with caution due to the small sample size and the non-probability sampling approach used. This study was initially conceived as a preliminary study due to resource and logistics limitations. There were two reasons for choosing JPSHC as our study site: It is the third largest primary government healthcare center in the country, and that it serves a sub-district with a sizable percentage of residents in the middle- to low-income groups. It is hoped that even at this preliminary stage, our study findings could encourage stakeholders to conduct similar and larger studies, using random sampling approach.”*  (c) Thank you for pointing this out. This study was originally carried out as a two-part study (one on the reasons for non-attendance questionnaire & another on self-sampling). However, we understand that it is irrelevant to mention this, and have therefore removed it entirely from the manuscript: in abstract (first sentence in Methods), and in discussion (first sentence). |
