# Supplementary material for: Reasons for non-attendance to cervical cancer screening and acceptability of HPV self-sampling among Bruneian women: A cross-sectional study
Source: PLoS One. 2022 Mar 14;17(3):e0262213. doi: 10.1371/journal.pone.0262213 (PMC8920207; doi:10.1371/journal.pone.0262213)
Supplement: Attachment — Submitted filename: JPSstudy_Rebuttal_Nov2021.docx [file pone.0262213.s009.docx]

**Point-to-point responses:**

| **Reviewer’s comments** | **Author’s Response** |
| --- | --- |
| Reviewer #2: great work! Continue to probe why older women will not screen after you do an educational intervention that shows the average age of cx ca is about 40 years old without any symptoms. | Thank you for your comment & advice! Noted with your suggestion and we shall try to continue with HPV research in Brunei. |
| Reviewer #3: General comments: Piloting self-testing in various cultural and geographic settings is important, however, I do not believe this adds to the body of literature supporting HPV self-testing. The sample size is also relatively small to make any meaningful comments about HPV prevalence; the authors do cite the need for additional research. The scope of this work would be more appropriate for a regional journal. | Firstly, a sincere thank you for your comments on this paper. It has indeed helped us to add clarifications and further streamline the reading flow, particularly the introduction and methods section.  While it is true that the sample size for HPV self-testing is small, we believe that this manuscript could contribute to the existing body of work on both HPV self-testing & on the reasons of not attending screening, in the local context. We hope that this extensive revision made could give a clearer picture in the Bruneian situation, and adds value to the global audience. |
| Non-attendees needs a clear definition somewhere, as well as clarification as to why a Pap test > 4 years ago was included (is this the recommendation in Brunei?). | We have added the definition of screening non attendees in the methods section:  “We recruited screening non-attendees, defined as married or ever married women between 20 and 65 years old and have never undergone cervical cancer screening, or did not have one within the recommended screening interval of 3 years”.  The recommended pap test screening interval in Brunei is 3 years. During data collection, we wanted to recruit those who have missed their next test. Four years was chosen mainly due to logistical reasons during data collection. To further clarify this, we have added the definition for non-attendee, as you have also suggested earlier. |
| Throughout the paper, you contrast HPV self swabs to a Pap test. I think the comparison you are really making is between a self-swab and a provider-collected test (using a pelvic examination). Or do you think there is a patient held belief in the reliability of a Pap test? Do you think it would be appropriate to rather compare HPV testing to any cervical cancer screening? Do you think there was any role of the desire to have a pelvic examination, other contexts have shown that patients feel more secure when a doctor as examined them. | Thank you for your comment. In this paper, we are actually comparing HPV testing with cervical cancer screening. The reason why we mentioned Pap test is because it is the only screening test we are currently using in Brunei (specifically liquid-based cytology). We realised that this is not been made clear & therefore have added this point in the introduction:  We do not think our patients distrust the reliability of the Pap test. Rather, we think that they may be more comfortable taking their own samples rather than "exposing" themselves to a healthcare provider. In Brunei, trained nurses generally perform the Pap test, with doctors performing in complicated cases (such as in menopausal women, women with 2 or more caesarean sections, and women with previous abnormal Pap test results). Also, pelvic examination is not done routinely, and is not necessary after a Pap test. |
| Specific comments: Abstract: Objective: specify <80% where? Explain non-attendance – do you mean non-attendance at recommended screening/screening intervals? | We have revised the indicate sentence as follows:  “Uptake for cervical cancer screening remains well below 80% as recommended by Brunei’s National Cervical Cancer Prevention and Control plan”. |
| Methods: describe the health center – tertiary referral center? You mention 4 years – is this the interval for screening in Brunei? You don’t mention the second survey here. Statistical analysis is not sufficient. | Thank you for your comments.  - The health center is actually a primary healthcare centre (we have clarified this in the methods section of both the abstract and main manuscript).  - The recommended screening interval in Brunei is 3 years. During data collection, we wanted to recruit those who did not attend during the 3-year period. Four years was chosen mainly due to logistical reasons during data collection. To further clarify this, we have added the definition for non-attendee, as you have also suggested earlier. |
| Results: it is hard for a reader to understand how you enrolled “non-attendees”. Perhaps that can simply be clarified. Need to specify in line 46 how many women accepted HPV self-swab. | We have added a sentence on the recruitment process: “This recruitment was done conveniently among women attending outpatient care and/or child health services at the primary healthcare center.”  We have revised the indicated sentence to add “among the 97 participants”. |
| Discussion: In the results you state that “Fear of bad results, embarrassment and lack of time” are the main causes for non-attendance, I’m not sure your suggested intervention directly tackles those issues. Perhaps specific education/community awareness that cervical cancer is a preventable disease? Line 52 is non-specific and needs clarification. | We have revised the following to make the suggested intervention more specific:  “Our findings suggest that promoting awareness on cervical cancer, clarifying any misconceptions of Pap test results, and highlighting that the disease is preventable and that early detection through screening can facilitate successful treatment would help increase screening uptake among Bruneian non-attendees. Response to HPV self-sampling was highly positive, suggesting the possibility of implementing this strategy in the local setting.” |
| Introduction: Generally not linear in its progression. First paragraph starts with effectiveness of screening and in particular screening with HPV testing. Second paragraph starts with more effective screening strategies for screening non-attendees. Third paragraph with specifies to Brunei. These are fine themes, but the authors lose focus as they add to these paragraphs. Also, I think there should be a more in-depth dive into screening non-attendance. What are patterns internationally and/or what are factors that cause non-attendance.  Specific feedback: Line 69 and 71: Screening non-attendees: what does this mean. How do you survey screening non-attendees? I assume there is a definition in methods, but some explanation is needed here. Lines 73-74: need full definition for CIN2+, you have only spelled out CIN Lines 74-79 seem to belong in the line of thinking of the first paragraph Lines 89-91 seem out of place and belong in the second paragraph Lines 95-97 make the statement about the importance/impact of this study more definitive. | Thank you for your comments. We have made quite extensive revisions to the introduction section and also re-arranged the sentences to improve the reading flow, following your suggestions.  We have also added here:  1. More details on the screening program in Brunei & the eligibility criteria.  2. Definition of CIN  3. A paragraph on the reasons for not attending screening. |
| Methods: The methods should be clear enough for someone else to repeat the study. It is not clear who was collecting the data, how issues of illiteracy were addressed (or was this an exclusion criteria). It is not stated where data was stored and analysis is not clear enough to be replicable. | Both questionnaires were initially designed to be self -administered. However, research assistant or trained nurse were present at most times to assist participants. The term “self -administered” has been added under the Data collection tool subsection.  Also, to be consistent with the terms used throughout the manuscript, we have revised the term “survey” to “questionnaire” in the abstract  It is already mentioned in the inclusion criteria that only those who can comprehend Brunei-Malay or English language will be included in the study.  We have provided the minimal dataset under supplementary information. For the analysis, please see our responses for your specific comments below (for results section). |
| Perhaps call this a pilot instead of “preliminary” | This has been revised, as suggested. |
| Line 115: not clear what “first part of the study is”  Line 117: how was second contact attempted? Why two weeks? I would suggest incorporating what was on the questionnaires with the first reference to the questionnaire, rather than having it as another section | Thank you for your suggestions. We have revised the section accordingly, and hope this revision further clarifies the whole recruitment procedure.  The second contact attempt was conducted via telephone call; this was clarified in the indicated sentence.  The 2 weeks interval was implemented to give sufficient time for the participant for read the information provided for the HPV self-sampling part, and also for logistical reasons (so that the assigned nurse can make the calls in batches). |
| Line 131: could not can | This has been revised, as requested |
| Line 173: It is not clear if frequencies were used to present the questions that were rated on a likert scale for the classifications described. If so, how were these frequencies compared (I notice the p-value in the results below). | Yes, frequencies were used. We have revised the indicated sentences as follows:  “Where appropriate, Mann-Whitney and/or Fisher’s exact tests were used to assess significant differences in sociodemographic characteristics, reasons for not attending screening and attitudes between women who agreed and women declined to proceed to the HPV self-sampling part of the study. Count responses for questions with the five-point Likert scale were categorized into three categories (agree, neutral, and disagree), and those with missing values were classified as neutral”. |
| Results: Line 195: drop “major” and “minor” qualifiers or define them. | We have revised the sentence to include the definitions in the methods section, as follows:  “From a prepared list of 16 possible reasons for not attending the screening program, participants were also asked to select one “Major” reason (defined as the main reason) and one or more “Minor” reasons (defined as other reasons for not attending the screening program).” |
| Line 219: what does this mean they agreed on the “severity of cervical cancer”? | Thank you for pointing this out. We have revised the sentence as follows:  “While the responses vary when asked about their health and susceptibility to disease, most agreed on the benefits of undergoing Pap test (92.0%, n = 160), and that cervical cancer is a severe and potentially lethal disease (82.8%, n = 144; Table 3 and S2 Table).” |
| Line 234, would avoid the use of subjective qualifiers | We have revised the sentence as follows:  “Among those who participated in self-sampling (55.7%, n = 97), their responses on Q2 (Table 4) were ~~generally positive and encouraging~~ mostly positive” |
| Line 248, It is pretty remarkable that 8/9 women with HPV had abnormal pap smears. Also does this mean only 1 participant had CIN3? What about the rest? | We have updated the follow-up results for all the 9 HPV positive women:  “The HPV 16 positive case was found to have high-grade ASC-US (ASCUS-H) on the initial follow up smear, but found negative after subsequent follow up cervical biopsy. Among the other 8 non-HPV 16/18 HPV genotype positive cases: 2 had negative follow up smears, 4 were reported to have ASC-US on their initial follow up smears, but had subsequent negative follow up smears, 1 was reported to have low-grade squamous intraepithelial lesion (LSIL) on her initial follow up smear, and had a subsequent negative follow up smear, and 1 was found to have CIN 3 with glandular involvement on the follow up smear and cervical biopsy, and has received treatment with a cone biopsy where excision of CIN 3 was confirmed.” |
| Discussion: First sentence can be eliminated. | This has been revised, as suggested. |
| Line 310 – this should be mentioned in the methods section in exclusion criteria of non-married women | This has been clarified in the definition of screening non-attendees. |
| Lines 272-274 – this hadn’t come up in the results, so I wouldn’t bring it into the discussion | For this sentence, the results actually did come out from our study. Specifically in Figure 2 & S1 Table. We have added “(Fig. 2 and S1 Table)” to further clarify this. |
| Lines 319 – 321 – rephrase this, don’t use “hope” to convey this sentiment | We have revised the indicated sentence as follows:  “However, even at this pilot stage, our study findings could encourage stakeholders to conduct similar and larger studies, using random sampling approach”. |
| Tables: Table 1: percentages are confusing because you don’t know how many women are in each age group, so you don’t know if the percentage is of the total number of women or the number in the age group. I think the percentages would be more understandable if they were the percentage of women in the age group who joined/declined. What’s the difference between “never” and “no” for HPV vaccination? | As suggested, we have revised Table 1 to show row percentages for participants who joined/declined HPV self-sampling. For consistency, Table S1 was also revised accordingly, to show row percentages for participants who were tested hr-HPV positive/negative.  The difference is that “never” means have never gotten HPV vaccine, and “no” means have not completed HPV vaccination. We have revised the wording in Table 1 to further clarify this. |
| Table 4, question 12 – do you think the wording of the question was leading to participants by putting “proper” before Pap test? | This sentence was phrased the same way as in the actual Q2 used during data collection. We opted to use the term “proper” to better distinguish between the “new” self-sampling that they have just conducted by themselves, and indicate to participants that we are asking about the standard screening test being used in Brunei (i.e. the Pap test).  As such, we do not think that the term “proper” in the sentence is leading to participants, rather the intention was to help participants distinguish between the 2 tests.  Please note that, during the time of data collection (and also as of November 2021), HPV self-sampling is not being routinely conducted in Brunei. |
